# Distinguishing active from quiescent disease in ANCA-associated vasculitis using attenuated total reflection Fourier-transform infrared spectroscopy

**DOI:** 10.1038/s41598-021-89344-8

**Published:** 2021-05-11

**Authors:** Adam D. Morris, Camilo L. M. Morais, Kássio M. G. Lima, Daniel L. D. Freitas, Mark E. Brady, Ajay P. Dhaygude, Anthony W. Rowbottom, Francis L. Martin

**Affiliations:** 1 Department of Renal Medicine, Royal Preston Hospital, Lancashire NHS Foundation Trust, Preston, UK; 2 School of Pharmacy and Biomedical Sciences, University of Central Lancashire, Preston, UK; 3 Institute of Chemistry, Biological Chemistry and Chemometrics, Federal University of Rio Grande Do Norte, Natal, Brazil; 4 Department of Immunology, Royal Preston Hospital, Lancashire NHS Foundation Trust, Preston, UK; 5 School of Medicine, University of Central Lancashire, Preston, UK; 6 Biocel UK Ltd, Hull, UK

**Keywords:** Biomarkers, Nephrology, Rheumatology, Computational biophysics

## Abstract

The current lack of a reliable biomarker of disease activity in anti-neutrophil cytoplasmic autoantibody (ANCA) associated vasculitis poses a significant clinical unmet need when determining relapsing or persisting disease. In this study, we demonstrate for the first time that attenuated total reflection Fourier-transform infrared (ATR-FTIR) spectroscopy offers a novel and functional candidate biomarker, distinguishing active from quiescent disease with a high degree of accuracy. Paired blood and urine samples were collected within a single UK centre from patients with active disease, disease remission, disease controls and healthy controls. Three key biofluids were evaluated; plasma, serum and urine, with subsequent chemometric analysis and blind predictive model validation. Spectrochemical interrogation proved plasma to be the most conducive biofluid, with excellent separation between the two categories on PC2 direction (AUC 0.901) and 100% sensitivity (F-score 92.3%) for disease remission and 85.7% specificity (F-score 92.3%) for active disease on blind predictive modelling. This was independent of organ system involvement and current ANCA status, with similar findings observed on comparative analysis following successful remission-induction therapy (AUC > 0.9, 100% sensitivity for disease remission, F-score 75%). This promising technique is clinically translatable and warrants future larger study with longitudinal data, potentially aiding earlier intervention and individualisation of treatment.

## Introduction

Anti-neutrophil cytoplasmic autoantibody (ANCA)-associated vasculitis (AAV) characterises an autoimmune disorder that results in inflammation and necrosis of small- and medium-sized blood vessels, causing potential multi-organ and life threatening disease. Central to its pathogenesis is the activation of primed neutrophils through the interaction of ANCA with myeloperoxidase (MPO) and proteinase-3 (PR3) target antigens expressed on their cell surface, resulting in neutrophil degranulation, endothelial damage and amplification loop that ensues^1–3^. Current immunosuppressive treatment strategies are effective with improved patient survival, but carry a significant risk of treatment-related toxicity and long-term patient morbidity that often results from the sequelae of relapsing disease and required re-exposure to therapy^4–6^. The lack of a reliable biomarker of disease activity to aid the early detection of relapsing disease represents a significant clinical challenge, risking potentially undertreated disease or over exposure to therapy.

Attenuated total reflection Fourier-transform infrared (ATR-FTIR) biospectroscopy offers a highly versatile, non-destructive and cost-effective means of analysing a given biological sample to determine its chemical composition; in effect providing a surrogate of its metabolomic profile. Within any sample, the chemical bonds that make up its constituent molecules will exhibit a periodic vibrational pattern. Those with a dipole moment are active within the infrared (IR) spectral range. ATR-FTIR exploits this by exposing a biological sample to mid-IR radiation to provide a spectral pattern based on the degree of IR absorption. As such, it can be used to quantitatively detect biochemical changes caused by pathology and provide a unique spectral fingerprint for disease states. Advancements in instrumentation and computational chemometric analysis have enabled ATR-FTIR to be applied to a wide range of biofluids and tissue samples, across numerous medical disciplines. Studies have employed it to detect disease with a high degree of sensitivity and specificity, including inflammatory arthropathy, neurodegenerative disease and malignancy^7–10^. Its use in the field of nephrology is emerging with tissue analysis of both native and transplanted kidneys to aid current histological assessment and prognostication^11–14^, detection of cast nephropathy^15^, renal stone analysis^16,17^ and more recently analysis of peritoneal and haemodialysis effluent^18,19^.

One previous study has applied ATR-FTIR spectroscopy in vasculitis, demonstrating promising results, with several characteristic spectral markers identified from urine in a rodent model of crescentic glomerulonephritis and patients with renal limited disease^20^. This phase one proof-of-concept study aims to determine the potential use of ATR-FTIR spectroscopy in AAV as novel biomarker of disease activity, through analysis of a range of biofluids and correlation with clinical parameters from patients with multisystem disease.

## Results

### Study population

One hundred and eight participants are included in the present study; 25 with active disease (AD), 38 in disease remission (DR), 10 with membranous nephropathy (MM), five with minimal change disease (MCD), 10 with immunoglobulin A nephropathy (IgA), 10 with pre-renal acute kidney injury (AKI) in the context of infection and 10 healthy controls (HC). Descriptive baseline characteristics for the active and remission disease cohorts are shown in Table 1. AAV participant flow through the study is shown in Supplementary Information (SI) Fig. S1. Amongst these two groups, overall mean age was 66 ± 11.9 years with no significant gender predominance. The majority were Caucasian, with only one South-Asian participant in the remission cohort. Baseline characteristics for the disease control groups are outlined in SI Table S1.

**Table 1:** Characteristics of study population at the time of enrolment.

	Active Disease (n = 25)	Disease Remission (n = 38)
**Mean Age (SD)**	64 ± 11.9	67 ± 11.9
**Sex**		
Male	12/25 (48%)	20/38 (53%)
Female	13/25 (52%)	18/38 (47%)
**Median serum creatinine (μmol/L)**	216 (347–132)	122 (174–94)
**Mean eGFR (mls/min/1.73m**^**2**^**)**	22 (48–8)	47 (65–29)
**Newly diagnosed disease**	20/25 (80%)	–
**Relapsing disease**	5/25 (20%)	–
**ANCA serotype**		
MPO	9/25 (36%)	6/38 (16%)
PR3	12/25 (48%)	14/38 (37%)
Negative	4/25 (16%)	18/38 (47%)
**BVAS**	16 ± 9.6	0
**Organ involvement**		
Constitutional signs or symptoms	15/25 (60%)	–
Mucous membranes/Ophthalmic	6/25 (24%)	–
Cutaneous	1/25 (4%)	–
ENT	12/25 (48%)	–
Respiratory	6/25 (24%)	–
Cardiovascular	1/25 (4%)	–
Gastrointestinal	0	–
Renal	18/25 (72%)	–
Neurological	5/25 (20%)	–
Multisystem disease	17/25 (68%)	–
Renal limited	4/25 (16%)	–
**Other laboratory salient laboratory results:**		
Mean Haemoglobin (g/L)	100 ± 28.3	130 ± 13.4
Mean White cell count (10^9^/L)	9 ± 3.7	7.2 ± 2.2
Mean Lymphocyte count (10^9^/L)	1.2 ± 0.7	1.3 ± 0.6
Mean Neutrophil count (10^9^/L)	7 ± 3.6	5.1 ± 2.2
Mean Platelet count (10^9^/L)	309 ± 143.5	270 ± 80.6
Median CRP (mg/L)	42 (64.8–4.8)	2.6 (5.3–1.2)
Median ESR (mm/hr)	42 (80.5–9)	12 (19.8–5)
Median ESR (mm/hr)	42 (80.5–9)	12 (19.8–5)
Mean serum albumin (g/L)	34.7 ± 7.3	44.4 ± 2.9
Mean serum total protein (g/L)	62 ± 9.8	67.1 ± 4.7
**Co-morbidities**		
Ischaemic heart disease	1 (4%)	4 (11%)
Congestive cardiac failure	0	1 (3%)
Cerebrovascular disease	1 (4%)	2 (5%)
Hypertension	5 (20%)	18 (47%)
Peripheral vascular disease	0	1(3%)
Diabetes Mellitus	2 (8%)	3 (8%)
Chronic pulmonary disease	6 (24%)	5 (13%)
Chronic liver disease	0	0
Connective tissue disease	1 (4%)*	0
Malignancy	2 (8%)**	1 (3%)***
**Immunosuppression**		
None	8 (26%)	7 (18%)
Prednisolone****	14 (56%)	15 (39%)
Methylprednisolone	7 (28%)	0
Rituximab within the preceding 6 months	1 (4%)	13 (34%)
Cyclophosphamide	2 (8%)	2 (5%)
Azathioprine	0	6 (16%)
Mycophenolate	0	4 (11%)
Methotrexate	0	1 (3%)

*eGFR* estimated glomerular filtration rate, *ANCA* anti-neutrophil cytoplasmic autoantibody, *MPO* myeloperoxidase, *PR3* proteinase-3, *ESR* erythrocyte sedimentary rate, *CRP* C-reactive protein.

*One case or rheumatoid arthritis in remission, **One case of non-metastatic prostate cancer in remission & one case of colonic tubular adenoma, ***One case of non-melanoma skin cancer, **** Amongst the remission cohort a daily dose of prednisolone ≥ 5 mg was considered significant.

Of those with active disease, 16% (n = 4) had undetectable circulating ANCA, 68% (n = 17) had multisystem disease and of the remaining 32% (n = 8) with single organ disease, 4 were renal limited, 3 were limited to ear nose and throat disease and one had ophthalmic disease. Amongst the remission cohort 52% (n = 20) had persisting positive ANCA serology despite clinically quiescent disease. Overall two patients died, both in the AD cohort and both due to infection. No patients were lost to follow up. Paired remission samples were attained from 14 patients in the AD cohort for comparative analysis following successful remission induction therapy. Amongst this group the majority (n = 13) were ANCA positive at initial diagnosis, of which six remained ANCA positive at the time of paired remission sample collection.

### ATR-FTIR: spectral data and classification models

ATR-FTIR spectrochemical interrogation of plasma samples yielded the highest degree of accuracy for discrimination between AD and DR, followed by serum and urine sample analysis respectively. Plasma sample data is presented below, with serum and urine datasets provided in the SI.

**Figure 1:**
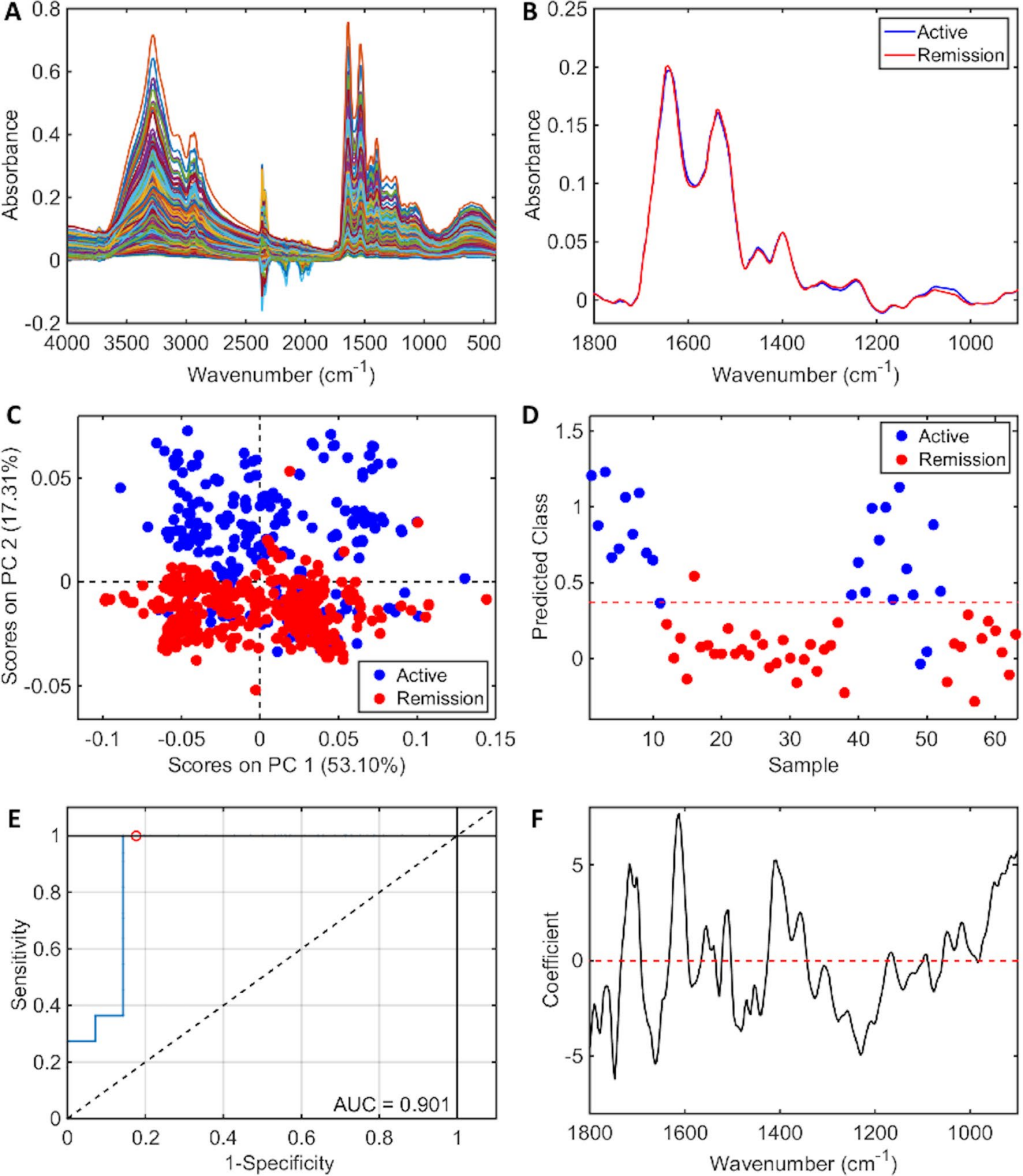
ATR-FTIR spectral classification of active disease vs. disease remission for plasma samples—(**A**) Raw spectral data, (**B**) Pre-processed spectra, (**C**) PCA scores plot, (**D**) PLS-DA discriminant function graph, (**E**) ROC curve for PLS-DA, (**F**) PLS-DA coefficients for identification of spectral biomarkers.

Figures 1a and 1b show the raw and pre-processed IR absorption spectra attained from plasma samples between the 900–1800 cm^−1^ spectral range for participants with AD and DR. Spectra initially appear to overlap. Given that a vast majority of constituent biomolecules present in plasma will be common to most individuals, this would be expected. Following second order differentiation to resolve overlapping peaks, multivariate analysis using principal component analysis (PCA) exhibits good separation between AD and DR on PC2 direction (Fig. 1c). A subsequent supervised classification model using partial least squares discriminant analysis (PLS-DA) was undertaken (Fig. 1d). In this process 60% of samples with known categories of either AD or DR were used as a training set to generate the classification model, with cross validation to prevent overfitting and blind assessment of the remaining 40% of samples to test the models predictive performance. The receiver operating characteristic (ROC) curve (Fig. 1e) demonstrates excellent ability of ATR-FTIR spectroscopy to distinguish between AD and DR using this classification system with an area under the curve of 0.901. This predictive classification model is shown in Table 2, with 100% sensitivity (F-score 92.3%) for DR and 85.7% specificity (F-score 92.3%) for correctly identifying AD.

**Table 2:** Classification parameters for plasma samples in active disease (AD) vs. disease remission (DR).

AD vs. DR	Accuracy (%)	Sensitivity (%)	Specificity (%)	F-Score (%)
Training (5 LVs)	93.6	96.3	90.9	93.5
Cross-validation	91.7	92.6	90.9	91.7
Test	92.8	100	85.7	92.3

**Figure 2:**
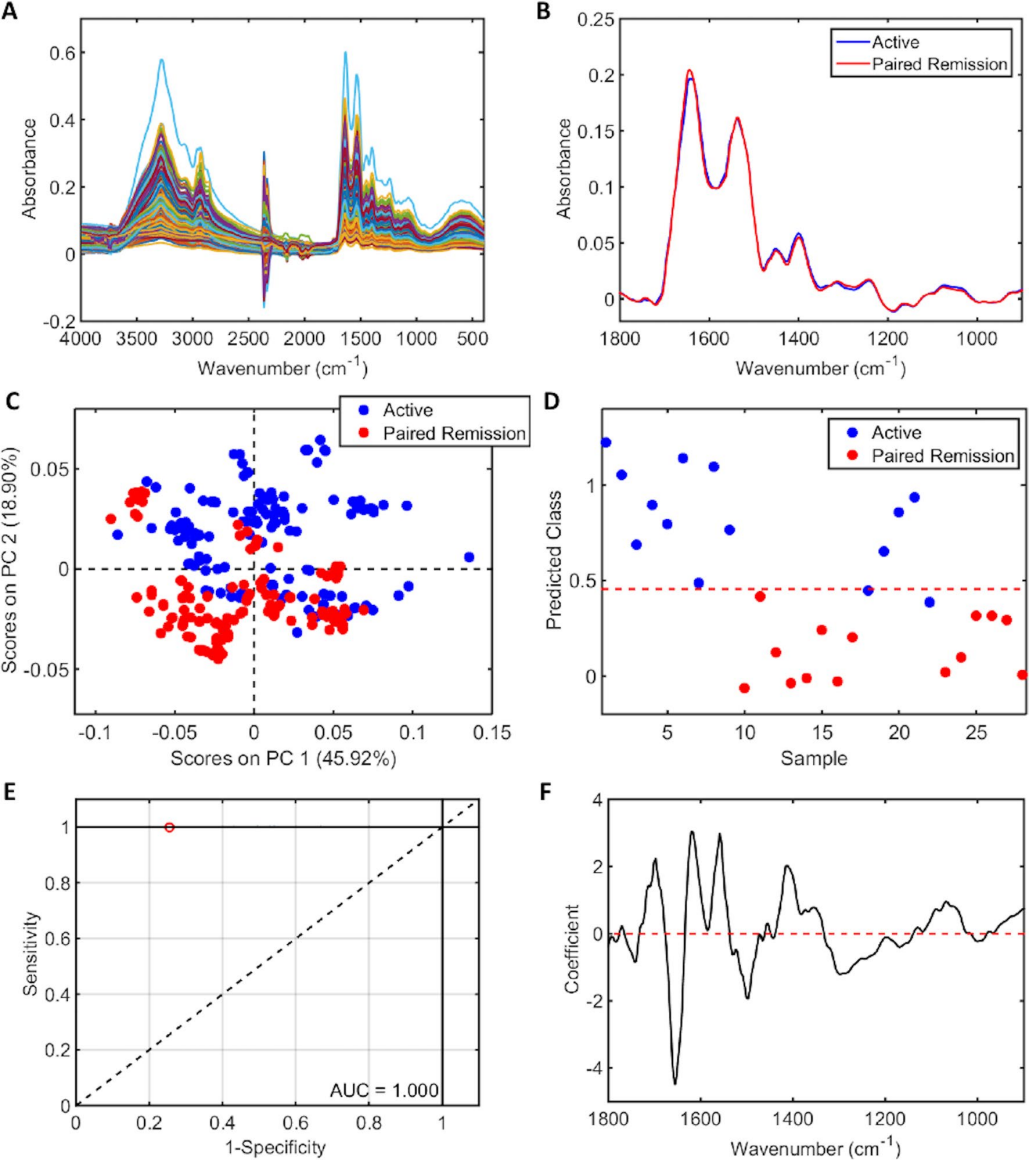
ATR-FTIR spectral classification of active disease vs. paired remission for plasma samples following successful remission induction therapy—(**A**) Raw spectral data, (**B**) Pre-processed spectra, (**C**) PCA scores plot, (**D**) PLS-DA discriminant function graph, (**E**) ROC curve for PLS-DA, (**F**) PLS-DA coefficients for identification of spectral biomarkers.

Following successful remission induction therapy, analysis of paired remission samples revealed similar findings with good ability to discriminate AD and DR; PLS-DA AUC > 0.9 (Fig. 2) and 100% sensitivity (F-score 75%) for DR on predictive modelling (Table 3)*.* There is a high level of accuracy in distinguishing AD from healthy controls (see SI, Fig. S2b,c and Table S2). On PCA scores of HC and DR, there was no clear segregation pattern between the two groups on PC2 direction following removal three outlier spectra from the HC cohort (see SI, Fig. S2e). Subsequent analysis of all participants in disease remission (n = 52), inclusive of those in remission at the time of study enrolment (n = 38) and paired remission samples (n = 14), confirmed excellent separation of spectral data from all control groups (see SI, Fig. S3e,f. and Table S3). There was equally good separation of AD from all control groups (see SI, Fig. S3b,c and Table S3).

**Table 3:** Classification parameters for plasma samples in active disease (AD) vs. paired remission (PR).

AD vs. PR	Accuracy (%)	Sensitivity (%)	Specificity (%)	F-Score (%)
Training (2 LVs)	100	100	100	100
Cross-validation	82.6	87.5	77.8	82.4
Test	80.0	100	60.0	75.0

### Correlation with clinical parameters

The correlation between ATR-FTIR spectral data for plasma and relevant clinical variables amongst those with AD is shown in Table 4. Based on the determination coefficient (R^2^), other than serum albumin and total protein, there is no demonstrable significant correlation shown and the ability of ATIR-FTIR spectroscopy to accurately discriminate between AD and DR was independent of organ system involvement, detectable circulating ANCA, ANCA titre in cases of seropositive disease, renal function, commonly used markers of inflammation and other salient laboratory results. There was no significant correlation between spectral data and BVAS, a validated and widely applied clinical assessment tool of disease severity. Correlation between ATR-FTIR spectral data for plasma and relevant clinical variables amongst those with DR is shown in SI Table S4. This confirmed no significant findings. The sensitivities and specificities provided in both tables only relates to the ability to separate the two groups. It was not possible to calculate these respective values for age, BVAS ANCA titre and other routine salient laboratory results, as these are variables with defined numerical values for which discriminatory algorithms could not be executed.

**Table 4:** Comparative analysis between clinical variables and ATR-FTIR spectral data from plasma samples.

Active disease	Sensitivity of clinical variable	Specificity of clinical variable	Coefficients of determination (R^2^)
**Age**	–	–	0.01
**Gender**	0.75	0.77	0.29
**BVAS**	–	–	0.19
**Organ involvement:**			
Constitutional signs or symptoms	0.60	0.60	0.00
Mucous Membrane/Ophthalmic	0.58	0.50	0.12
Cutaneous	0.83	1.00	0.02
ENT	0.39	0.67	0.14
Respiratory	0.58	0.50	0.02
Cardiovascular	1.00	1.00	0.00
Renal	1.00	0.94	0.52
Neurological	0.50	0.20	0.04
**ANCA Positivity**	0.67	0.75	0.06
**ANCA Serotype**			
MPO	0.44	0.50	0.00
PR3	0.67	0.38	0.01
Negative	0.75	0.71	0.02
**ANCA titre**	–	–	0.12
**Serum creatinine (μmol/L)**	–	–	0.27
**eGFR(mls/min/1.73m**^**2**^**)**	–	–	0.45
**Haemoglobin**	–	–	0.51
**White cell count**	–	–	0.51
**Lymphocyte count**	–	–	0.24
**Neutrophil count**	–	–	0.52
**Platelet count**	–	–	0.08
**CRP**	–	–	0.18
**ESR**	–	–	0.29
**Serum albumin**	–	–	0.86
**Total protein**	–	–	0.65

*ENT* ear nose and throat, *ANCA* anti-neutrophil cytoplasmic autoantibody, *MPO* myeloperoxidase, *PR3* proteinase-3, *BVAS* Birmingham vasculitis activity score, *eGFR* estimated glomerular filtration rate, *ESR* erythrocyte sedimentary rate, *CRP* C-reactive protein.

Treatment data at the time of sample collection is outlined in Table 1. The varied distribution of therapy provided the opportunity to tentatively determine the impact of immunosuppression on spectral analysis independently of the effects of disease activity. Of the 56% (n = 14) of patients in the AD cohort and 39% (n = 15) in the DR cohort on prednisolone, the median daily dose was 40 mg (IQR 60–20) and 5 mg (IQR 10–5) respectively. Neither dosing regimens accounted for any difference in data variance (see SI, Figs. S4 and S5). Similarly, rituximab exposure up to six months prior to sample collection did not influence spectral analysis in the DR cohort (see SI, Fig. S6). Meaningful subgroup analysis evaluating the impact of methylprednisolone, cyclophosphamide, azathioprine, methotrexate and mycophenolate were not feasible owing to the limited sample size of each subgroup.

### Key spectral biomarkers

**Figure 3:**
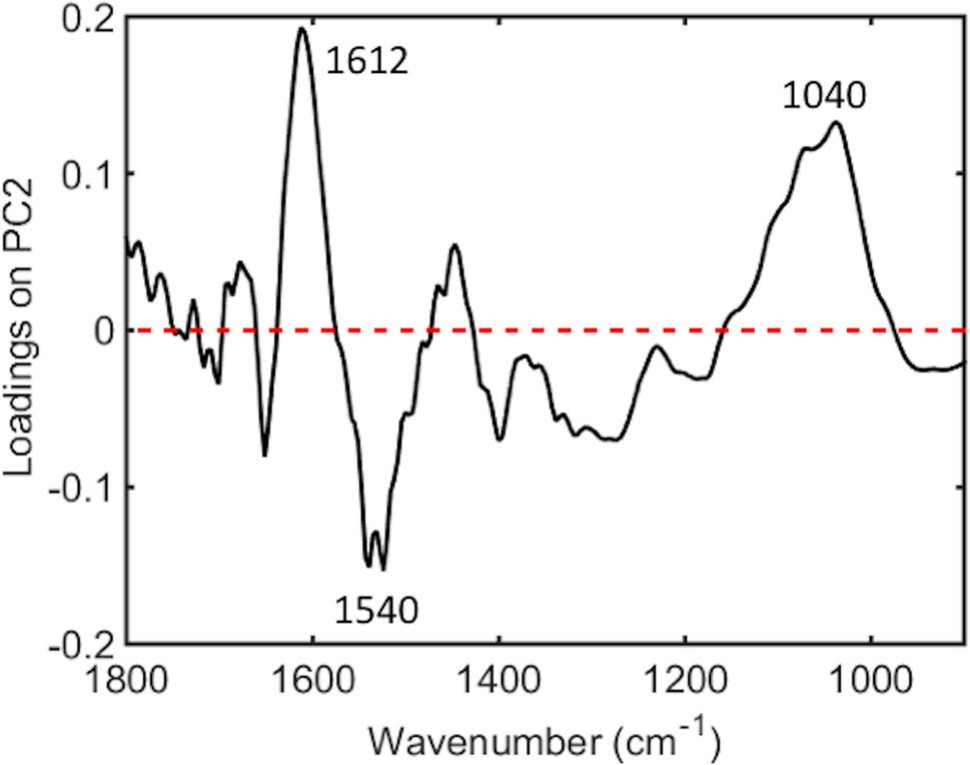
Main band differences for healthy controls (HC) vs. active disease (AD) using PCA loadings on PC2 from plasma samples—1612 cm^−1^ (higher in AD, adenine vibration in DNA), 1540 cm^−1^ (higher in HC, protein Amide II β-sheet), 1040 cm^−1^ (higher in AD, symmetric PO2^−^ stretching in RNA/DNA).

Key distinguishing peaks can be identified amongst the spectral data in each model based on PLS-DA coefficients. The wavenumber-variables responsible for largest between group differences provide biomarker extraction through the chemical bond they represent, which in turn can be associated with a particular cellular activity. Amongst AD and HC cohorts, wavenumber-variables 1612 cm^−1^ (adenine vibration in DNA) and 1040 cm^−1^ (symmetric PO_2_^−^ stretching in RNA/DNA) were both higher in AD, whereas 1540 cm^−1^ (protein Amide II β-sheet) was higher in HC (Fig. 3).

Notable wave-number variables characterising AD from DR and their potential corresponding chemical bonds are outlined in Table 5. Noteworthy peaks for AD were in the 1620 – 1716 cm^−1^ range, representing nucleobase functional group expression as the main contributors; 1620 cm^−1^ (base carbonyl stretching and ring breathing mode of nucleic acids), 1698 cm^−1^ (C_2_=O guanine), 1701 cm^−1^ (*ν*(C=O) thymine) and 1716 cm^−1^ (*ν*(C=O) DNA/RNA). Lipid (1748 cm^−1^, 1778 cm^−1^) and protein functional groups at the Amide I (1662 cm^−1^) and Amide II (1481 cm^−1^) bands were associated with disease remission.

**Table 5:** Potential spectral biomarkers for distinguishing active disease and disease remission using plasma samples based on the PLS-DA coefficients (*ν* = stretching; *δ* = bending).

Wavenumber (cm^−1^)	Tentative assignment	Influence on AD
1778	*ν*(C = C) lipids	↓
1748	*ν*(C = C) lipids	↓
1716	*ν*(C = O) DNA/RNA	↑
1701	*ν*(C = O) thymine	↑
1662	Amide I	↓
1509	In-plane *δ*(CH) phenyl ring	↑
1481	Amide II	↓
1408	*δ*(CH_3_)	↑
1358	*ν*(C–O), *δ*(CH), *δ*(NH)	↑
1230	*ν*_as_(PO_2_^−^)	↓
948	Phosphodiester region (collagen and glycogen)	↑
914	Phosphodiester region (collagen and glycogen)	↑
1698	C_2_ = O guanine	↑
1654	C = O, C = N, N–H of adenine, thymine, guanine, cytosine	↓
1620	Base carbonyl stretching and ring breathing mode of nucleic acids	↑
1558	Ring base mode	↑
1415	CH deformation	↑

## Discussion

The present study provides the first evidence that ATR-FTIR spectroscopy has the potential to provide an accurate biomarker of active disease and treatment response in multisystem AAV. To our knowledge this is the first study of its kind, in which we demonstrate its use as a quantitative method to distinguish active from quiescent disease with a high degree of sensitivity and specificity from plasma. This was applicable both in renal and extra-renal disease irrespective of current ANCA serology.

Given the inherent procedural risks, serial renal biopsies for histological confirmation of active disease is not practical and in the context of extra-renal disease, a tissue biopsy is often not feasible and typically has a low diagnostic yield^21^. Current approaches in clinical practice use two main biomarkers to help predict potential relapse; ANCA and B-cell population, however their clinical utility remains limited. Persisting ANCA positivity, ANCA reappearance and anti-PR3 associated disease have been associated with a higher rate of relapse^22–25^, but despite this a significant proportion of de-novo and relapsing disease occurs in the absence of detectable circulating ANCA^26–30^, particularly amongst patients with extra-renal disease^31^. The significance of rising titres also remains debateable^32,33^. Moreover, ANCA positivity has been shown to occur in healthy individuals^34–36^ as well as other systemic illnesses^37^, further restricting its use. Similarly, while B-cell repopulation following targeted therapy with rituximab has been associated with relapsing disease^22,38,39^, follow up data from other large trials has not corroborated this finding^33,40^ and active disease has been shown to occur with B-cell activity in tissue despite depletion in serum^41^. Amongst other potential biomarker tools, urinary soluble CD163 (sCD163) and urinary monocyte chemoattractant protein-1 (MCP-1) have both demonstrated significant promise, with higher levels of both associated with active ANCA-associated glomerulonephritis^42–47^. Furthermore, combination of the two has yielded a high specificity and positive likelihood ratio for relapsing disease^48^. However, any potential role of urinary sCD163 and MCP-1 remains limited to renal vasculitis, with a robust non-invasive biomarker of multisystem disease still lacking. Other potential biomarkers including novel autoantibodies, autoantigen gene expression, serum cytokines and degradation products of the alternative complement pathway have either failed to be validated or require further investigation^49^.

Biospectroscopy is an innovative candidate for the development of a functional biomarker of disease activity. Yu et al.used ATR-FTIR spectroscopy to analyse urine samples from rodent models of inflammatory glomerulonephritis, as well as a limited number of patients with ANCA positive pauci-immune glomerulonephritis to determine renal inflammation and injury^20^. Several key characteristic spectral markers were identified that correlated with the progression and severity of disease. In particular, both the 1545 cm^−1^ and 1033 cm^−1^ wavenumber intensity correlated with disease severity in the rodent model, with normalisation to baseline following treatment with dexamethasone. However, the 1545 cm^−1^ band intensity increased with declining renal function amongst both patients with active disease and those in remission, failing to discriminate between the two groups. The 1545 cm^−1^ band was also present in the murine model of lupus nephritis, suggesting that it may not be specific to vasculitis, instead reflecting glomerular inflammation and damage. Nonetheless, these original findings demonstrated the potential application of ATR-FTIR spectra from urine as marker of disease activity in patients with ANCA positive renal limited disease.

In order to detect differences between patients with active disease and those in clinical remission, we employed ATR-FTIR to extract spectral data from three key biofluids; plasma, serum and urine. Using unsupervised learning where spectra are classified without any prior sample knowledge, overall category separation using PCA was good in both plasma and serum samples. On subsequent blind predictive modelling of known and unknown spectral profiles with PLS-DA, our findings demonstrated that plasma was the most accurate biofluid for discriminating between the two categories, correctly identifying active disease in 85.7% of cases and 100% of those in remission. This finding was independent of ANCA serology with 16% of patients in the AD cohort having undetectable circulating ANCA and 53% of patients having persistent ANCA positivity despite disease remission. Parallel findings were also seen in the AD cohort where paired remission samples were attained; demonstrating that not only can ATR-FTIR spectroscopy be used as a biomarker of active disease, but it could also be applied help determine treatment response. Our results were also applicable to both renal and non-renal disease with the majority of patients in the AD cohort having multisystem disease and no demonstrable correlation of discriminating spectral data with organ system involvement. The lack of any significant correlation between the spectral data and currently used clinical markers including ANCA, CRP and ESR is unsurprising as the latter are all known to have a limited association with disease activity. Similarly, the absence of any significant correlation with BVAS suggests that the application of ATR-FTIR spectroscopy may only be used to identify active disease and not disease severity. Group separation on PCA plot of spectral data attained from urine may have been restricted as not all included patients had renal involvement and of those who did, a urine sample could not be attained from three patients.

Several key wavenumber-variables associated with active disease from plasma samples were of particular interest, namely 1620 cm^−1^, 1698 cm^−1^, 1701 cm^−1^ and 1716 cm^−1^ which are all associated increased nucleic acid expression. This may reflect the known genetic contribution to disease susceptibility and epigenetic factors of disease activity, with reduced DNA methylation of MPO and PRTN3 resulting in increased autoantigen expression and disease activity^50–52^. Alternatively, recognising the role of nuclear extracellular traps (NETs) in disease pathogenesis, this this finding may simply reflect increased free cell DNA as a result of NET remnants and apoptotic cells^53–56^. Protein groups at the Amide I (1662 cm^−1^) and Amide II (1481 cm^−1^) bands were associated with disease remission. This may be a reflection of plasma protein abundance in the acute phase of illness with slightly lower trend in serum albumin observed in active disease (34.7 ± 7.3 g/L) compared to disease remission (44.4 ± 2.9 g/L). However, the determination coefficient (R^2^) for both serum albumin and total protein did not confirm a positive correlation with spectral data amongst the disease remission cohort, but trended towards significance in active disease leaving the relevance of this result unclear. Mean serum total protein was largely similar between the two groups; 62 ± 9.8 g/L vs. 67.1 ± 4.7 g/L. Mass spectrometric analysis could be applied in future study to help correlate the ATR-FTIR spectral pattern with potential key compositional properties.

The translation of ATR-FTIR spectroscopy into clinical practice is feasible. Portable handheld devices are currently in use in non-medical fields^57^. Coupled with the integration of chemometric algorithms, minimal sample requirements and label free preparation means that ATR-FTIR spectroscopy offers a potential low cost, fast, automated near-patient test to complement current clinical practice and help identify patients with active disease. Although our results are encouraging they should be considered within the context of its primary limitation; despite taking measures to avoid the risk of overfitting, the risk of bias from insufficient training data remains a potential factor in this phase one study for biomarker discovery. A larger future phase two validation study is required to provide sufficient longitudinal training data and prevent bias from small sample size. Based on known outcomes, machine-learning using forward feature extraction algorithms could then be used to construct prediction models based on extracted spectral features. Such features would also lend novel insight into evolving mechanisms of action. We calculate that the optimal sample size for validation would be 199 samples (79 active and 120 remission cases) for a power of 80%. For the optimal sample size we used a Fisher’s exact test considering the spectral proportion of active and remission cases, which is roughly 0.4:0.6 (see SI, Figure S15). Given the rare nature of disease, such a timely study would be feasible using existing biobanks from previous large randomised control trials in the field, with samples from various disease time points alongside current standard diagnostic methods. A second limitation to consider is the control groups used in the present study. Although good separation of active disease from all control groups was seen, other than AKI with systemic infection, disease control groups were otherwise restricted to renal limited pathology. Any future study of ATR-FTIR as a biomarker of multisystem AAV would benefit from inclusion of controls with other systemic inflammatory conditions, such as systemic lupus erythematous or rheumatoid arthritis to help evaluate its clinical utility further.

The absence of a functional biomarker that accurately correlates with disease activity in AAV represents a significant clinical need. Our findings demonstrate that ATR-FTIR spectroscopy offers a novel means of distinguishing between active disease and remission in multisystem disease, independent of ANCA status. As well as aiding diagnosis, this may facilitate early intervention and tailored maintenance therapy to help improve patient outcomes, particularly in seronegative disease. These findings require validation in a larger study with longitudinal data and comparison against a wider range of glomerular and autoimmune conditions.

## Materials and methods

### Patients and ethics

Over a fourteen-month period from February 2019 to March 2020, paired blood and urine samples were collected from consecutive patients with active AAV and those in disease remission. The active disease cohort consisted of patients with new presentation or relapsing disease. The definition of AAV as outlined by the 2012 Chapel Hill Consensus Conference was used. As the histological confirmation of disease is often not a viable diagnostic tool in the context of extra-renal disease and a reference standard test with a sufficient degree of sensitivity and specify is lacking in such cases, the index test of ATR-FTIR spectroscopy was evaluated in the context of this widely used criterion as reference standard for clinical diagnosis. Patients who did not meet this criterion, who were aged < 18 years, unable to provide consent or exhibited dual positivity with anti-glomerular basement membrane disease were excluded. Disease remission was defined as a Birmingham Vasculitis Activity Score (BVAS) of 0. A significant difference in the spectral data amongst patients with and without active AAV was the primary outcome of interest. As this study is applying a novel and previously untested technology to this patient group a sample size could not be calculated. All participants were registered with the Department of Renal Medicine regional vasculitis service at Lancashire Hospitals NHS Foundation Trust, UK. Informed written consent was obtained prior to enrolment in accordance with study approval from the Health Research Authority, Cambridge South Research Ethics Committee (REC reference 18/EE/0194) and the Research and Development team in the Centre for Health Research and Innovation at Lancashire Teaching Hospitals NHS Foundation Trust. All experiments were carried out in accordance with the relevant guidelines and regulations.

The following clinical data was collected at baseline assessment; demographics, clinical presentation, BVAS and salient laboratory results including current ANCA serotype were applicable, serum creatinine, haemoglobin, white cell count, lymphocyte count, neutrophil count, platelet count, C-reactive protein erythrocyte sedimentary rate and urine protein creatinine ratio. Urine samples were sent for microscopy and culture to determine the presence of bacteriuria and its potential impact as a confounding factor on ATR-FTIR sample analysis. For patients with active disease, further samples were collected following successful remission induction therapy where possible for comparative analysis. Control groups including membranous nephropathy, minimal change disease, immunoglobulin A nephropathy, acute kidney injury in the context of infection and healthy individuals were included for analysis. Participants in the healthy control group were not known to have renal impairment and had a normal dipstick urinalysis.

### Sample collection and preparation

Samples from participants with active disease were taken in both the outpatient clinic and acute in-patient setting. All remission and disease control samples were taken in the outpatient clinic. Healthy control samples were attained from individuals working in the hospital outside of the laboratory setting. Whole blood samples were collected in EDTA and serum separator tubes. All blood and urine samples were centrifuged at 3000 rpm, 4 °C for 10 min. The resulting plasma, serum and supernatant urine were collected in 0.5 ml Eppendorf tubes and stored on site at − 80 °C. When required for experimentation samples were thawed at room temperature, after which 30 μl aliquots were placed on IR-reflective aluminium coated FisherBrand slides and left to air dry for a minimum of 2 h prior to spectroscopic analysis.

### ATR-FTIR spectral acquisition

ATR-FTIR spectra were attained using a Bruker Tensor 27 FTIR spectrometer will Helios ATR attachment (Bruker Optics Ltd, Coventry UK), operated by OPUS 6.5 software. The sample area was defined by the diamond crystal internal reflective element, approximately 250 μm by 250 μm. Spectra were acquired from 10 locations on each sample; five central and five peripheral to help minimise any potential bias. Parameters for spectral acquisition consisted of 32 scans per location, 8 cm^−1^ spectral resolution with 2 × zero-filling and 6 mm aperture setting, yielding a data spacing of 4 cm^−1^ over 4000–400 cm^−1^ spectral range. The diamond crystal was cleaned with distilled water, dried with Kimwipes and a background absorption spectra was taken at the start of each new sample analysis to account for atmospheric conditions.

### Spectral pre-processing

The spectral data were imported into MATLAB R2014b environment (MathWorks Inc., USA) for pre-processing and subsequent multivariate analysis. Pre-processing consists of mathematical techniques employed to the raw spectral data to remove or reduce contributions of signals that are not related to the analyte target property or to the sample discrimination, hence, reducing chemically irrelevant variations in order to improve the accuracy and precision of qualitative and quantitative analyses^58^. Herein, the raw spectral data were pre-processed by automatic weighted least squares (AWLS) baseline correction and vector normalisation^59^. Finally, prior to model construction by partial least squares discriminant analysis (PLS-DA), the pre-processed data are mean-centred.

### Multivariate analysis

Multivariate classification by means of PLS-DA was employed to discriminate the spectral data based on the experimental classes. Firstly, the pre-processed data were split into training and test (external validation) sets using the MLM algorithm^60^. The training set, composed of 60% of the samples, was used for model construction, whose optimisation step (defining the number of latent variables (LVs) for PLS-DA) was performed via venetian blinds cross-validation. The remaining samples (40% of the dataset) assigned to the test set were used to evaluate the model classification performance. The spectral replicas per sample were averaged prior to model construction so the model was constructed on a sample-basis, hence, with no overlap of samples between the training and test sets.

PLS-DA is a well-known chemometric technique of supervised classification. It is based on a linear classification model for which the classification criterion is obtained by partial least squares (PLS)^61^. In PLS-DA, PLS is applied to the pre-processed data, reducing them to a few number of LVs, in which the category variables for each class in the training set is used to optimise the model. Then, a straight line that divides the classes’ spaces is delineated^62^. In addition to PLS-DA, prior to supervised classification, the pre-processed data also underwent an exploratory analysis by principal component analysis (PCA) in order to identify possible natural clustering patterns or trends in the data through the analysis of the 2D PCA scores plots on principal components (PCs) 1 and 2^63^.

### Model validation

Model validation was performed by the calculation of accuracy, sensitivity, specificity and F-scores for the test set. The accuracy represents the total number of samples correctly classified, considering true and false negatives. The sensitivity represents the proportion of positive samples correctly classified, the specificity represents the proportion of negative samples correctly classified and the F-score measures the model performance considering imbalanced data^64^.

### Correlation with clinical variables

Correlation between the pre-processed spectra and individual clinical variables was evaluated by PLS regression (for continuous variables) and PLS-DA (for categorical variables). PLS and PLS-DA models were built using cross-validation. The association between spectra and a clinical variable was evaluated by assessing the determination coefficient (R^2^) and root mean square error of cross-validation (RMSECV). Clinical parameters for which the R^2^ was low, or RMSECV elevated, were considered to have poor correlation with the spectral data.

## Supplementary Information

Supplementary Information

## Data Availability

The authors declare that the data supporting the findings of this study are available within the paper and its supplementary information. The MATLAB code and instructions on how to process the data are presented in previous publications^49–51^.
