# Supplementary material for: Distinguishing active from quiescent disease in ANCA-associated vasculitis using attenuated total reflection Fourier-transform infrared spectroscopy
Source: Sci Rep. 2021 May 11;11:9981. doi: 10.1038/s41598-021-89344-8 (PMC8113456; doi:10.1038/s41598-021-89344-8)
Supplement: Supplementary file 1 — Supplementary Information [file 41598_2021_89344_MOESM1_ESM.pdf]

Supplementary Information

Distinguishing active from quiescent disease in ANCA-associated vasculitis using attenuated total reflection Fourier-transform infrared spectroscopy

Adam D. Morris[1]*, Camilo L. M. Morais[2], Kássio M. G. Lima[3], Daniel L. D. Freitas[3], Mark E. Brady[1], Ajay P. Dhaygude[1], Anthony W. Rowbottom[4, 5,] Francis L. Martin[6]*

[1]Renal Medicine, Royal Preston Hospital, Preston, UK
[2]School of Pharmacy and Biomedical Sciences, University of Central Lancashire, Preston, UK
[3]Institute of Chemistry, Biological Chemistry and Chemometrics, Federal University of Rio Grande do Norte, Natal, Brazil
[4]Department of Immunology, Royal Preston Hospital, Preston, UK
[5]School of Medicine, University of Central Lancashire, Preston, UK
[6]Biocel Ltd, Hull HU10 7TS, UK

No of Pages = 15
Number of Figures = 15
Number of Tables = 14

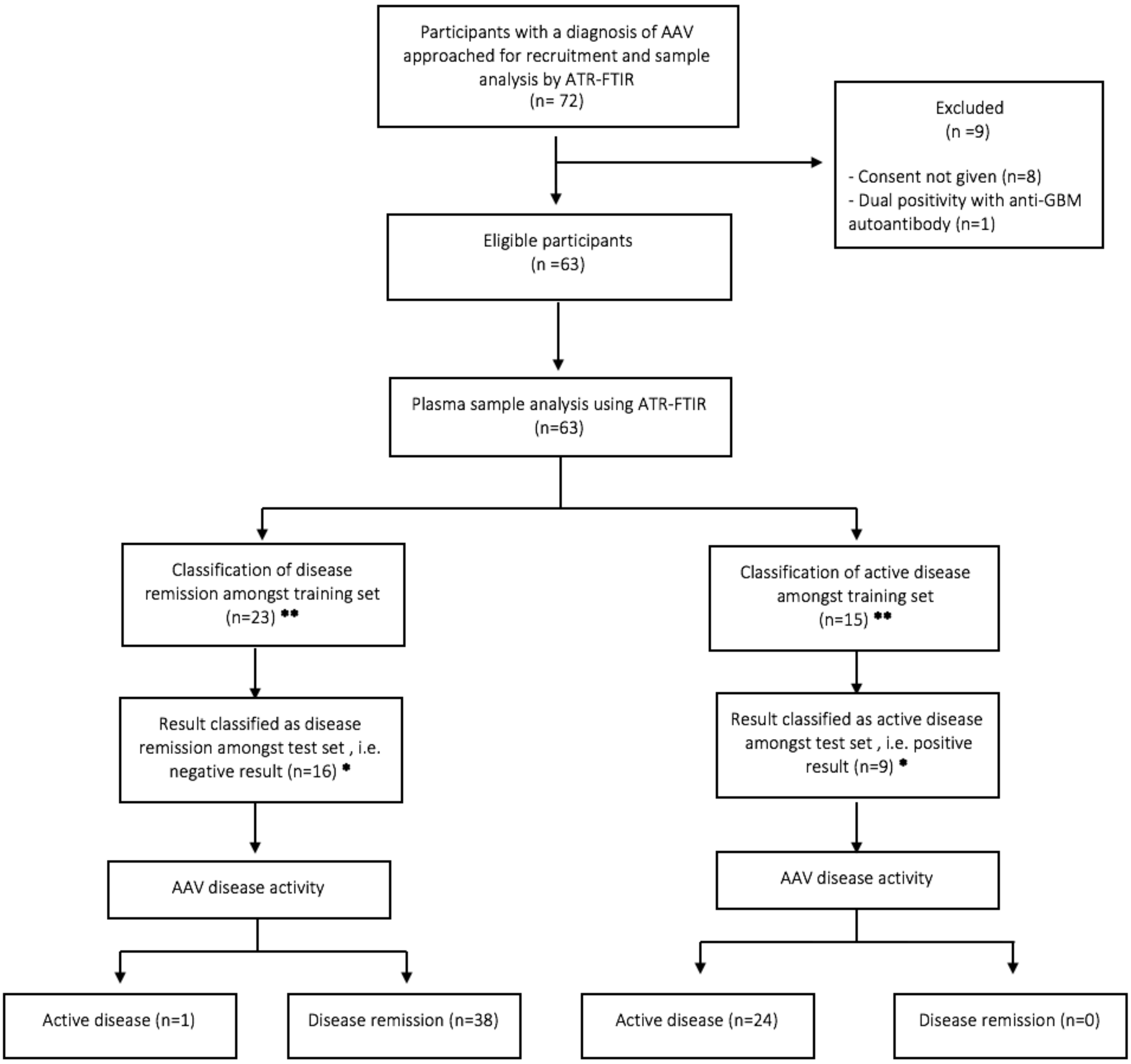

Figure S1: ANCA-associated **vasculitis participant flow -** AAV: ANCA-associated vasculitis, ***** Test set – samples used for blind predictive modelling for external validation of the classification systems performance, ******Training set – samples used for model construction of classification system

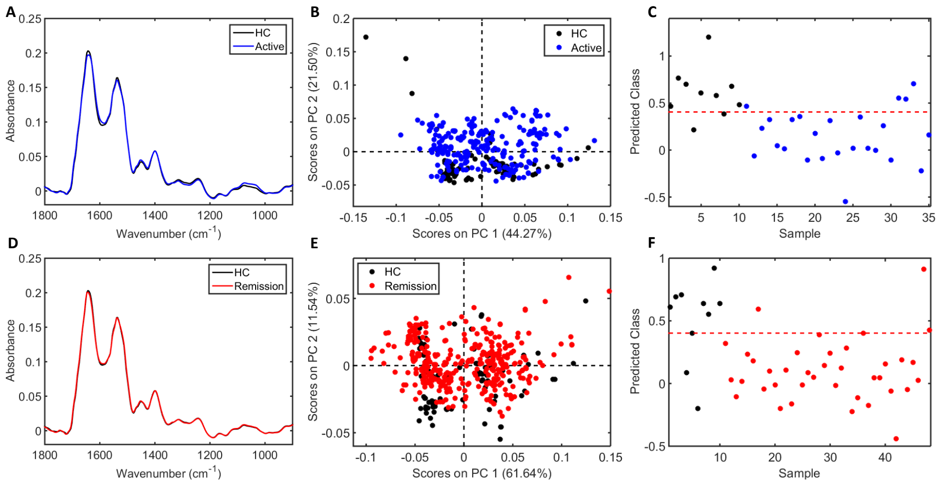

Figure S2: ATR-FTIR spectral **classification of healthy controls (HC) vs. active disease (AD) & healthy controls (HC) vs. disease remission (DR) for plasma samples – (A)** Average pre-processed spectral points for HC (n=100) & patients with AD (n=250) **(B)** PCA scores plot for HC & AD **(C)** PLS-DA discriminant function graph for classification of HC & AD using cross validation **(D)** Average pre-processed spectral points for HC (n=100) & DR (n=380) **(E)** PCA scores plot for HC & DR **(F)** PLS-DA discriminant function graph for classification of HC & DR using cross validation

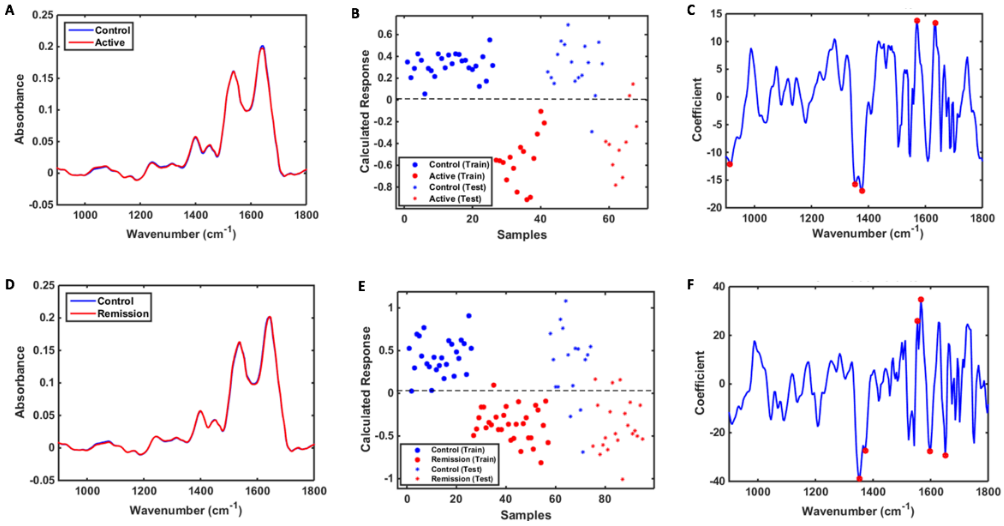

Figure S3: ATR-FTIR spectral **classification of control groups (CG) vs. active disease (AD) & control groups (CG) vs. disease remission (DR) for plasma samples. CG included healthy controls and disease controls of membranous nephropathy, minimal change disease, immunoglobulin A nephropathy and acute kidney injury with infection. The DR cohort consisted of those in disease remission at the time of enrolment (n=38) in addition to those who achieved disease remission post enrolment following successful remission induction therapy (n=14) – (A)** Average pre-processed spectral points for CG (n=450) & patients with AD (n=250) **(B)** PLS-DA discriminant function graph for classification of CG & AD using cross validation **(C)** PLS-DA coefficients for identification of main band differences for CG vs. AD **(D)** Average pre-processed spectral points for CG (n=450) & patients with DR (n=520) **(E)** PLS-DA discriminant function graph for classification of CG & DR using cross validation **(F)** PLS-DA coefficients for identification of main band differences for CG vs. DR

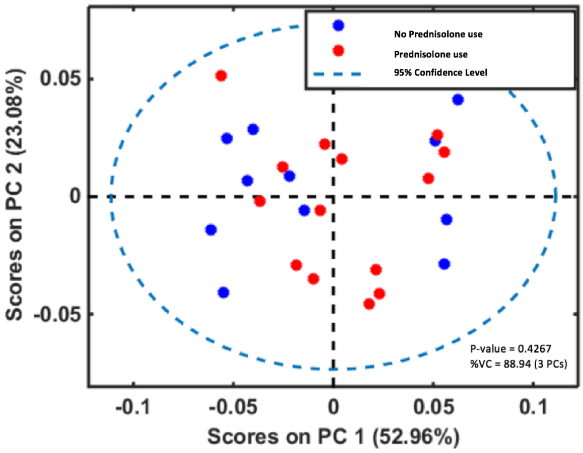

Figure S4: PCA scores plot of prednisolone use (n=14) vs. no prednisolone (n=11) use amongst the active disease cohort

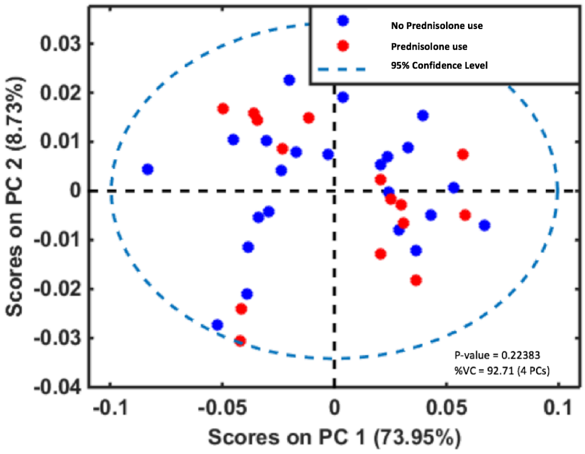

Figure S5: PCA scores plot of $\geq$ 5mg/day prednisolone use (n=15) vs. no prednisolone use (n=23) amongst the disease remission cohort

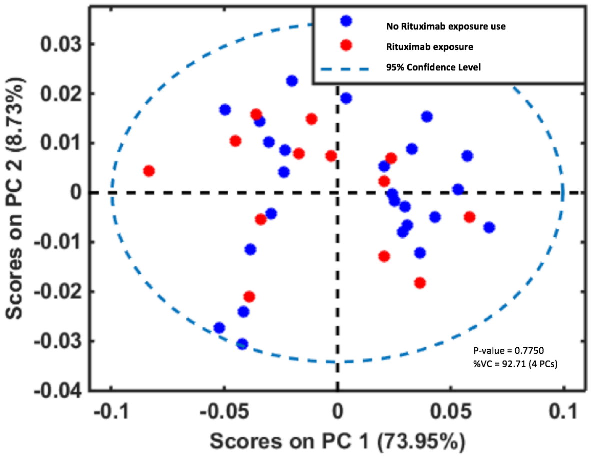

Figure S6: PCA scores plot of Rituximab exposure (n=13) vs. no Rituximab exposure (n=25) in the preceding 6 months amongst the disease remission cohort

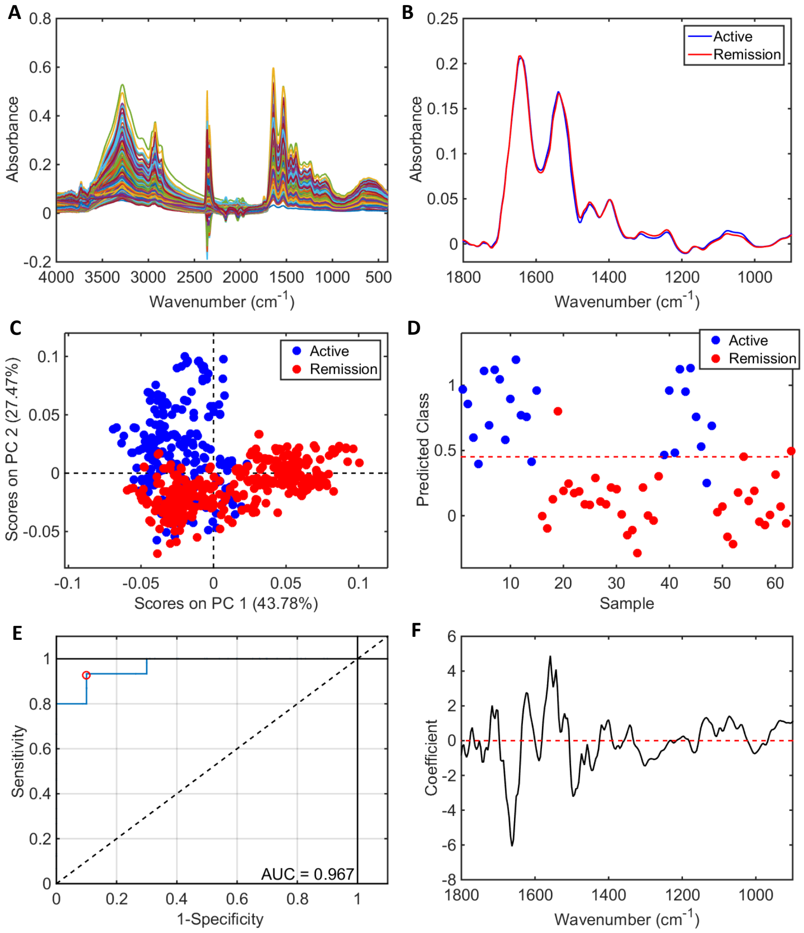

Figure S7: ATR-FTIR spectral **classification of active disease vs. disease remission for serum samples - (A)** Raw spectral data **(B)** Pre-processed spectra **(C)** PCA scores plot **(D)** PLS-DA discriminant function graph **(E)** ROC curve for PLS-DA **(F)** PLS-DA coefficients for identification of spectral biomarkers

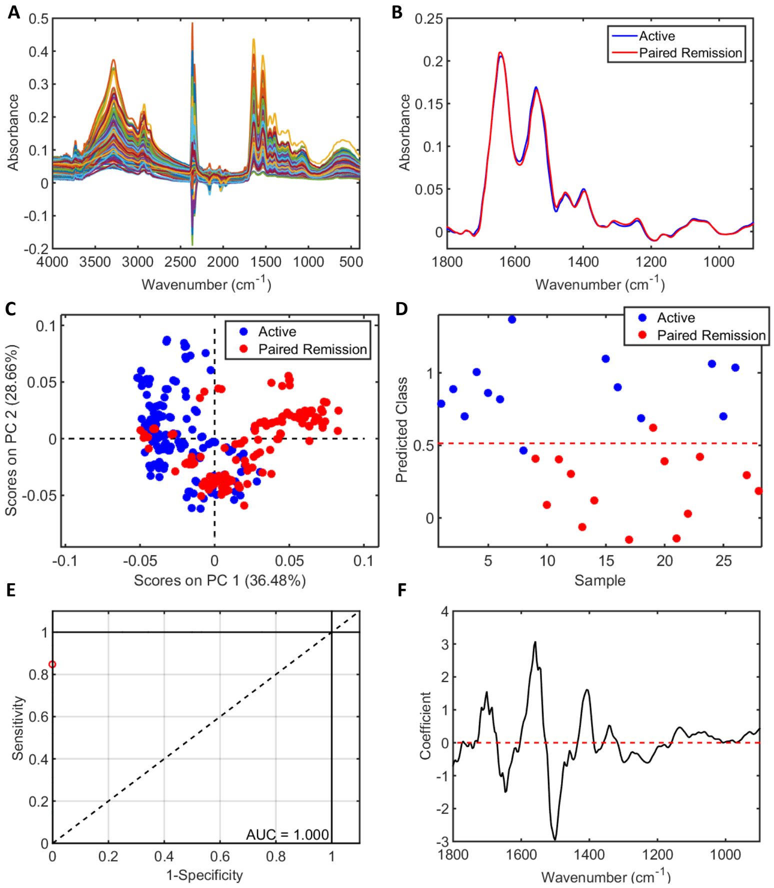

Figure S8: ATR-FTIR spectral **classification of active disease vs. paired remission for serum samples following successful remission induction therapy** - **(A)** Raw spectral data **(B)** Pre-processed spectra **(C)** PCA scores plot **(D)** PLS-DA discriminant function graph **(E)** ROC curve for PLS-DA **(F)** PLS-DA coefficients for identification of spectral biomarkers

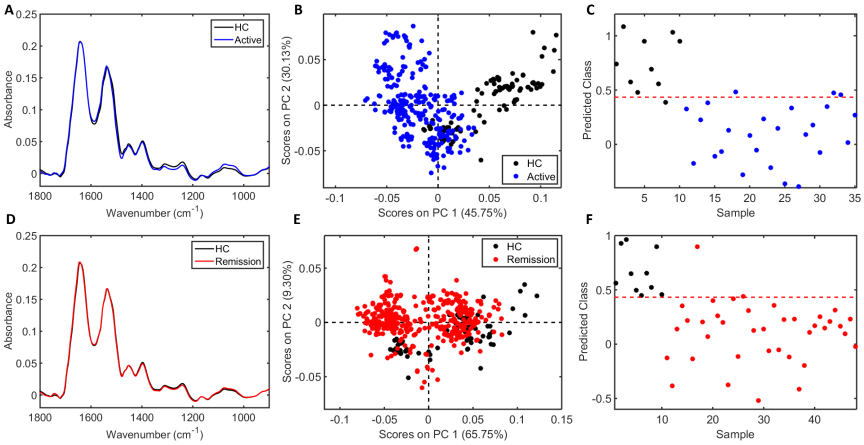

Figure S9: ATR-FTIR spectral **classification of healthy controls (HC) vs. active disease (AD) & healthy controls (HC) vs. disease remission (DR) for serum samples – (A)** Average pre-processed spectral points for HC (n=100) & patients with AD (n=250) **(B)** PCA scores plot for HC & AD **(C)** PLS-DA discriminant function graph for classification of HC & AD using cross validation **(D)** Average pre-processed spectral points for HC (n=100) & DR (n=380) **(E)** PCA scores plot for HC & DR **(F)** PLS-DA discriminant function graph for classification of HC & DR using cross validation

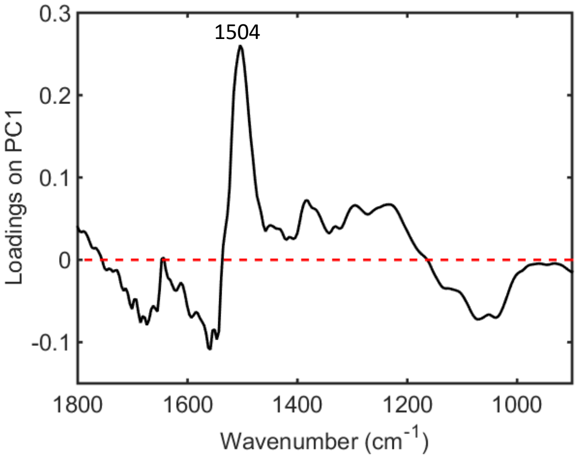

Figure S10: Main band differences for healthy controls (HC) vs. active disease (AD) using PCA loadings on PC2 from serum samples - 1504 cm^{-1} (higher in HC, Amide II).

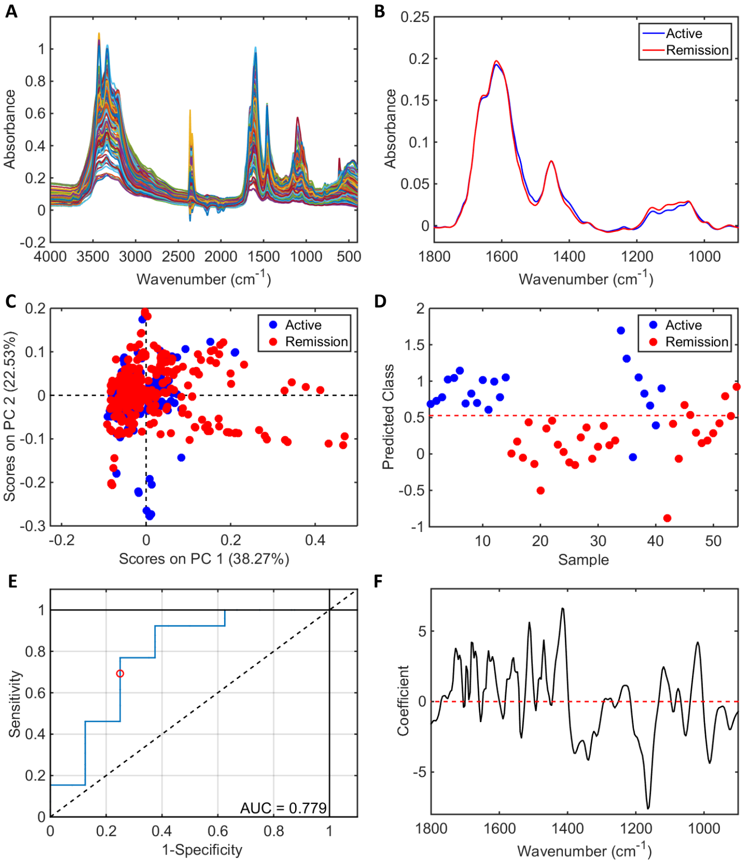

Figure S11: ATR-FTIR spectral **classification of active disease vs. disease remission for urine samples - (A)** Raw spectral data **(B)** Pre-processed spectra **(C)** PCA scores plot **(D)** PLS-DA discriminant function graph **(E)** ROC curve for PLS-DA **(F)** PLS-DA coefficients for identification of spectral biomarkers

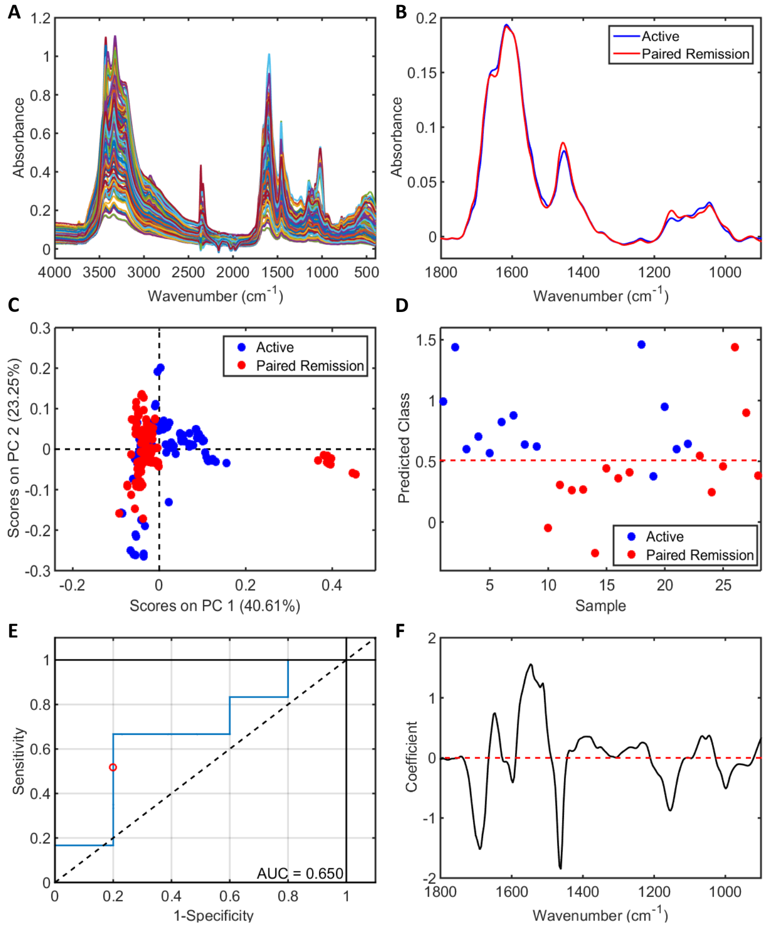

Figure S12: ATR-FTIR spectral **classification of active disease vs. paired remission for urine samples following successful remission induction therapy** - **(A)** Raw spectral data **(B)** Pre-processed spectra **(C)** PCA scores plot **(D)** PLS-DA discriminant function graph **(E)** ROC curve for PLS-DA **(F)** PLS-DA coefficients for identification of spectral biomarkers

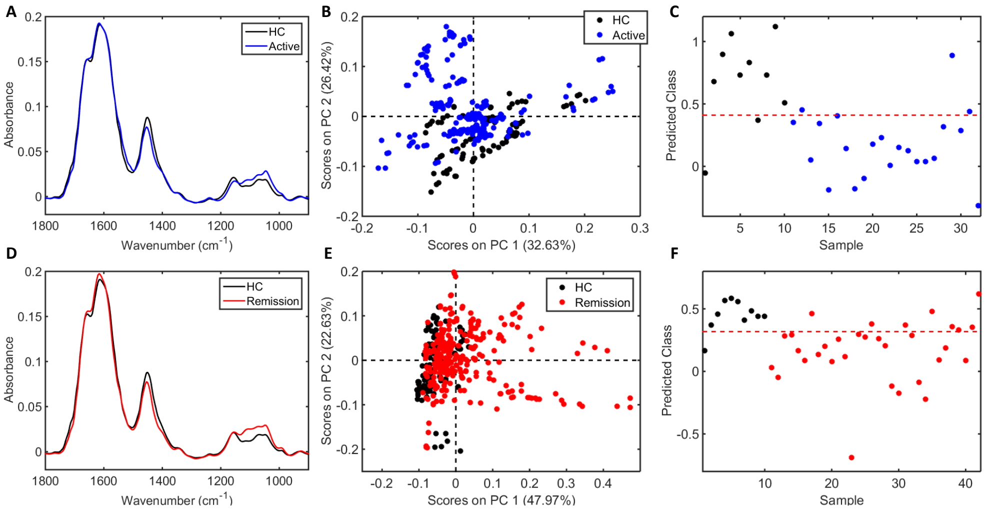

Figure S13: ATR-FTIR spectral **classification of healthy controls (HC) vs. active disease (AD) & healthy controls (HC) vs. disease remission (DR) for urine samples – (A)** Average pre-processed spectral points for HC (n=100) & patients with AD (n=220) **(B)** PCA scores plot for HC & AD **(C)** PLS-DA discriminant function graph for classification of HC & AD using cross validation **(D)** Average pre-processed spectral points for HC (n=100) & DR (n=320) **(E)** PCA scores plot for HC & DR **(F)** PLS-DA discriminant function graph for classification of HC & DR using cross validation

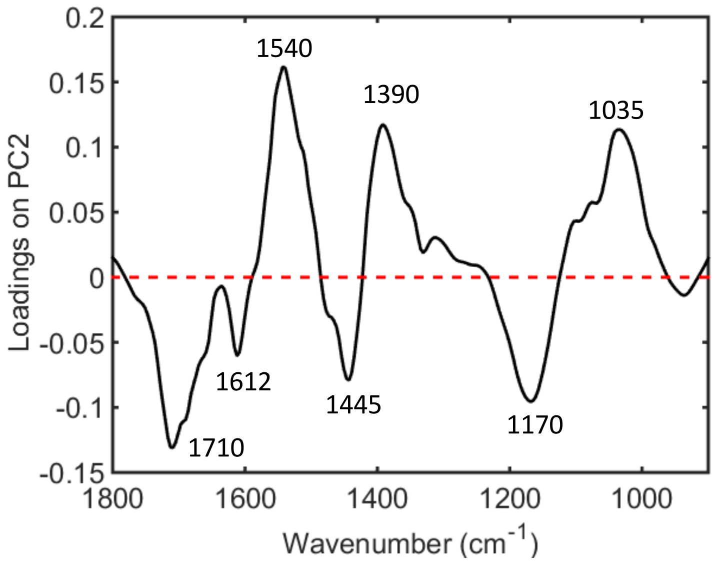

Figure S14: Main band differences for healthy controls (HC) vs. active disease (AD) using PCA loadings on PC2 from urine samples - 1710 cm^{-1} (higher in HC, C=O thymine), 1612 cm^{-1} (higher in HC, adenine vibration in DNA), 1540 cm^{-1} (higher in AD, protein amide II absorption β-sheet), 1445 cm^{-1} (higher in HC, δ(CH2) in lipids or fatty acids), 1390 cm^{-1} (higher in AD, CH3 bending), 1170 cm^{-1} (higher in HC, ν_{as}(CO-O-C)), 1035 cm^{-1} (higher in AD, skeletal *trans* ν(C-C) of DNA).

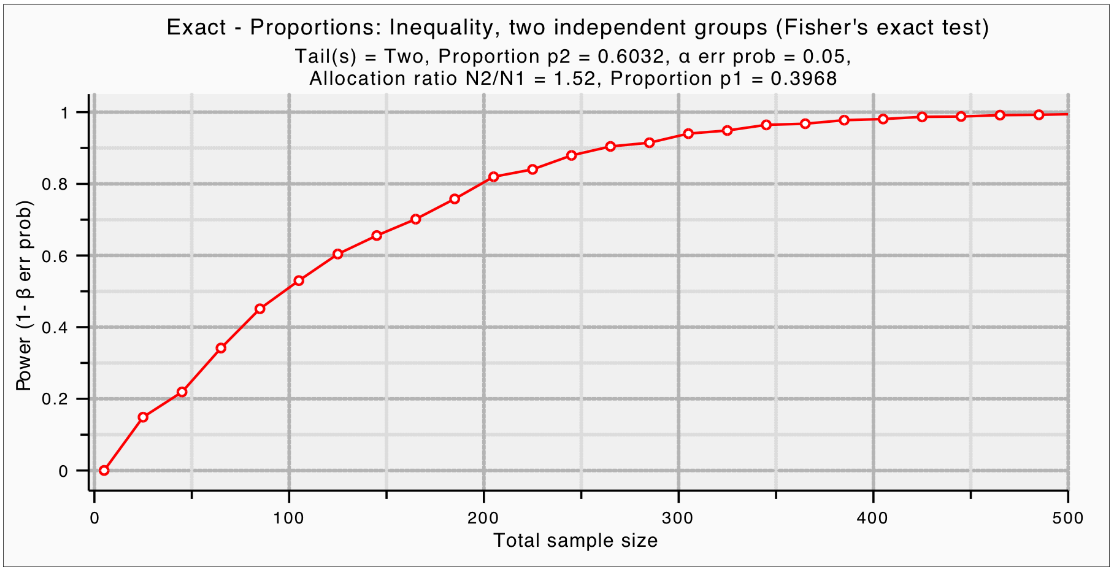

Figure S15: Power test based on a Fisher's exact test (two-tails, error probability = 0.05) showing the power varying the total sample size of active and remission cases.

Table S1: Characteristics of disease control groups at the time of enrolment & sample collection				
	MM (n=10)	MCD (n=5)	IgA (n=10)	AKI (n=10)
**Mean Age (SD)**	63 ± 9.4	50 ± 21.9	48 ± 12.9	71 ± 8.7
**Sex**				
Male	8	2	8	6
Female	2	3	2	4
**Median serum creatinine (µmol/L)**	103 (181-84)	81 (137-72)	212 (258-109)	330 (365-285)
**Median eGFR (mls/min/1.73m²)**	59 (60-32)	90 (90-35)	27 (74-22)	13 (14-12)
**Other Laboratory Salient Laboratory Results:**				
Mean Haemoglobin (g/L)	121 ± 12.9	133 ± 14.2	128 ± 17.9	93 ± 13
Mean White cell count (10^9/L)	6 ± 2.2	9 ± 4.2	7 ± 2.4	8 ± 4.2
Mean Lymphocyte count (10^9/L)	1.7 ± 0.6	1.8 ± 0.6	1.7 ± 0.5	0.9 ± 0.5
Mean Neutrophil count (10^9/L)	4 ± 1.6	6 ± 4.3	5 ± 2.2	6 ± 3.8
Mean Platelet count (10^9/L)	258 ± 84.7	291 ± 13.7	260 ± 49.7	253 ± 95
Median CRP (mg/L)	*	*	*	83 (122-47)

MM, Membranous Nephropathy; MCD, Minimal Change Disease; IgA, Immunoglobulin A Nephropathy; AKI, Acute Kidney Injury

Table S2: Classification parameters for plasma samples in healthy controls (HC) vs. active disease (AD) and disease remission (DR)				
	Accuracy (%)	Sensitivity (%)	Specificity (%)	F-Score (%)
HC vs. AD				
Training (4 LVs)	94.0	88.0	100	93.6
Cross-validation	82.0	84.0	80.0	82.0
HC vs. DR				
Training (3 LVs)	92.3	94.7	90.0	92.3
Cross-validation	81.0	92.1	70.0	79.5

Table S3: Classification parameters for plasma samples for control groups (CG) vs. active disease (AD) and all disease remission (DR)				
	Accuracy (%)	Sensitivity (%)	Specificity (%)	F-Score (%)
CG vs. AD				
Training (7 LVs)	100	100	100	100
Cross-validation	93	93	92	92
Test	89	80	94	86
CG vs. DR				
Training (9 LVs)	98	97	100	98
Cross-validation	86	87	85	86
Test	84	86	82	84

Table S4: Comparative analysis between clinical variables and ATR-FTIR spectral data from plasma samples			
Disease Remission	Sensitivity of clinical variable	Specificity of clinical variable	Coefficients of determination (R^2)
Age	-	-	0.03
Gender	0.55	0.61	0
ANCA Serotype			
MPO	0.61	0.2	0.06
PR3	0.21	0.8	0.02
Negative	0.67	0.53	0.01
ANCA titre	-	-	0.05
Serum creatinine (µmol/L)	-	-	0.43
eGFR(mls/min/1.73m^2)	-	-	0.3
Haemoglobin	-	-	0.26
White cell count	-	-	0.01
Lymphocyte count	-	-	0
Neutrophil count	-	-	0
Platelet count	-	-	0.01
CRP	-	-	0
ESR	-	-	0.16
Serum albumin	-	-	0.1
Total Protein	-	-	0.45

Table S5: Classification parameters for serum samples in active disease (AD) vs. disease remission (DR)				
AD vs. DR	Accuracy (%)	Sensitivity (%)	Specificity (%)	F-Score (%)
Training (4 LVs)	91.2	95.7	86.7	91.0
Cross-validation	91.2	95.7	86.7	91.0
Test	88.3	86.7	90.0	88.3

Table S6: Classification parameters for serum samples in active disease (AD) vs. paired remission (PR)				
AD vs. PR	Accuracy (%)	Sensitivity (%)	Specificity (%)	F-Score (%)
Training (2 LVs)	95.0	100	90.0	94.7
Cross-validation	95.0	100	90.0	94.7
Test	92.8	85.7	100	92.3

Table S7: Comparative analysis between clinical variables and ATR-FTIR spectral data from serum samples			
Active disease	Sensitivity of clinical variable	Specificity of clinical variable	Coefficients of determination (R^2)
**Age**	-	-	0.15
**Gender**	0.75	0.69	0.24
**BVAS**	-	-	0.13
**Organ involvement:**			
Constitutional signs or symptoms	0.75	0.40	0.24
Mucous Membrane / Ophthalmic	0.50	0.58	0.00
Cutaneous	0.92	1.00	0.02
ENT	0.33	0.31	0.23
Respiratory	0.83	0.63	0.03
Cardiovascular	1.00	1.00	0.01
Renal	1.00	1.00	0.54
Neurological	0.40	0.65	0.00
**ANCA Positivity**	0.91	0.75	0.22
**ANCA Serotype**			
MPO	0.33	0.81	0.00
PR3	0.75	0.54	0,00
Negative	0.75	0.86	0.22
**ANCA titre**	-	-	0.06
**Serum creatinine (µmol/L)**	-	-	0.28
**eGFR(mls/min/1.73m^2)**	-	-	0.44
**Haemoglobin**	-	-	0.54
**White cell count**	-	-	0.01
**Lymphocyte count**	-	-	0.21
**Neutrophil count**	-	-	0.04
**Platelet count**	-	-	0.15
**CRP**	-	-	0.28
**ESR**	-	-	0.00

ENT, ear nose and throat; ANCA, anti-neutrophil cytoplasmic autoantibody; MPO, myeloperoxidase; PR3, proteinase-3; BVAS, Birmingham vasculitis activity score; eGFR, estimated glomerular filtration rate; ESR, erythrocyte sedimentary rate; CRP, C-reactive protein

Table S8: Classification parameters for serum samples in healthy controls (HC) vs. active disease (AD) and disease remission (DR)				
	Accuracy (%)	Sensitivity (%)	Specificity (%)	F-Score (%)
**HC vs. AD**				
Training (3 LVs)	100	100	100	100
Cross-validation	89.0	88.0	90.0	89.0
**HC vs. DR**				
Training (3 LVs)	98.7	97.4	100	98.7
Cross-validation	97.3	94.7	100	97.3

Table S9: Potential spectral biomarkers for distinguishing active disease and disease remission using serum samples based on the PLS-DA coefficients (ν = stretching; δ = bending)		
Wavenumber (cm^{-1})	Tentative assignment	Influence on Active AAV
1716	ν(C=O) DNA/RNA	↑
1704	ν(C=O) thymine	↑
1662	Amide I	↓
1623	Base carbonyl stretching and ring breathing mode of nucleic acids	↑
1558	Ring base	↑
1543	Amide II	↑
1495	ν(C=C), δ(C-H)	↓
1701	C=O guanine	↑
1646	Amide I	↓
1558	Ring base mode	↑
1500	Amide II	↓
1407	CH3 asymmetric deformation	↑

Table S10: Classification parameters for urine samples in active disease (AD) vs. disease remission (DR)				
AD vs. DR	Accuracy (%)	Sensitivity (%)	Specificity (%)	F-Score (%)
Training (7 LVs)	100	100	100	100
Cross-validation	82.3	78.9	85.7	82.2
Test	72.1	69.2	75.0	72.0

Table S11: Classification parameters for urine samples in active disease (AD) vs. paired remission (PR)				
AD vs. PR	Accuracy (%)	Sensitivity (%)	Specificity (%)	F-Score (%)
Training (2 LVs)	100	100	100	100
Cross-validation	75.7	62.5	88.9	73.4
Test	65.0	50.0	80.0	61.5

Table S12: Comparative analysis between clinical variables and ATR-FTIR spectral data from urine samples

Active disease	Sensitivity of clinical variable	Specificity of clinical variable	Coefficients of determination (R^2)
Age	-	-	0.01
Gender	0.7	0.3	0.00
BVAS	-	-	0.17
Organ involvement:			
Constitutional signs or symptoms	0.2	0.6	0.10
Mucous Membrane / Ophthalmic	0.4	0.4	0.03
Cutaneous	0.8	1.0	0.05
ENT	0.6	0.7	0.12
Respiratory	0.3	0.8	0.00
Cardiovascular	1.0	0.9	0.00
Renal	0.7	0.4	0.01
Neurological	0.2	0.9	0.01
ANCA Positivity	0.7	0.6	0.05
ANCA Serotype			
MPO	0.5	0.9	0.15
PR3	0.9	0.3	0.01
Negative	0.6	0.6	0.04
ANCA titre	-	-	0.05
Serum creatinine (µmol/L)	-	-	0.02
eGFR(mls/min/1.73m²)	-	-	0.02
Haemoglobin	-	-	0.00
White cell count	-	-	0.24
Lymphocyte count	-	-	0.00
Neutrophil count	-	-	0.32
Platelet count	-	-	0.06
CRP	-	-	0.41
ESR	-	-	0.24
uPCR	-	-	0.46
Urine white cell count	-	-	0.01
Bacterial growth			
No growth (n=19)	0.8	0.0	0.01
Streptococcus agalactiae (n=1)	1.0	0.9	0.00
Enterococcus faecalis (n=1)	1.0	0.9	0.01
Mixed growth (n=1)	1.0	0.9	0.00

ENT, ear nose and throat; ANCA, anti-neutrophil cytoplasmic autoantibody; MPO, myeloperoxidase; PR3, proteinase-3; BVAS, Birmingham vasculitis activity score; eGFR, estimated glomerular filtration rate; ESR, erythrocyte sedimentary rate; CRP, C-reactive protein; uPCR, urine protein creatinine ratio; bacterial growth n=3

Table S13: Classification parameters for urine samples in healthy controls (HC) vs. active disease (AD) and disease remission (DR)				
	Accuracy (%)	Sensitivity (%)	Specificity (%)	F-Score (%)
HC vs. AD				
Training (3 LVs)	92.7	95.5	90.0	92.7
Cross-validation	85.4	90.9	80.0	85.1
HC vs. DR				
Training (1 LVs)	84.0	78.1	90.0	83.6
Cross-validation	85.6	81.3	90.0	85.4

Table S14: Potential spectral biomarkers for distinguishing active disease and disease remission using urine samples based on the PLS-DA coefficients (ν = stretching; δ = bending)

Wavenumber (cm^{-1})	Tentative assignment	Influence on Active AAV
1728	ν(C=O)	↑
1680	Amide I	↑
1632	ν(C=C) uracil	↑
1512	In-plane δ(CH) phenyl ring	↑
1470	$\delta(CH_2)$ methylene chains in lipids	↑
1415	δ(C-H), δ(NH), ν(C-N)	↑
1380	$\delta(CH_3)$	↓
1339	Collagen	↓
1164	ν(C-O) of C-OH groups of serine, threosine and tyrosine of proteins	↓
1020	DNA	↑
984	OCH_3 polysaccharides	↓
1689	Base carbonyl stretching and ring breathing mode of nucleic acids	↓
1647	Amide I	↑
1546	Amide II of proteins	↑
1512	In-plane CH bending from phenyl rings	↑
1460	$\delta_{as}(CH_3)$ collagen	↓
1155	C-O stretching	↓
